# Changes of NADH Fluorescence from the Skin of Patients with Systemic Lupus Erythematosus

**DOI:** 10.1155/2019/5897487

**Published:** 2019-12-24

**Authors:** Jaroslaw Bogaczewicz, Kamila Tokarska, Anna Wozniacka

**Affiliations:** Department of Dermatology and Venereology, Medical University of Lodz, Poland 90-647 Lodz, Plac J. Hallera 1/6, Łódź, Poland

## Abstract

**Introduction:**

The blood circulation of the skin is an accessible and representative vascular bed for examining the mechanisms of microcirculatory function. Endothelial function is impaired in systemic lupus erythematosus (SLE), which implies disorders in cell metabolism dependent on blood circulation; however, noninvasive monitoring of metabolism at the tissue and cell level is absent in daily clinical practice.

**Objective:**

The aim of the study was to examine changes of NADH fluorescence from the epidermis of a forearm measured with the flow mediated skin fluorescence (FMSF) technique in patients with SLE and to investigate whether they are associated with clinical manifestation of the disease.

**Materials and Methods:**

The study enrolled 36 patients with SLE and 34 healthy individuals. Changes of NADH fluorescence were measured using FMSF on the forearm in response to blocking and releasing of blood flow. The results were represented as ischemic (IR max and IR auc) and hyperemic response maximum and area under the curve (HR max and HR auc).

**Results:**

IR max, IR auc, HR max, and HR auc were all lower in patients with SLE (*p* < 0.05) compared with controls. All four parameters were negatively correlated (*p* < 0.05) with patient age. No difference was found in NADH fluorescence between SLE patients with malar rash, discoid rash, photosensitivity, oral ulcers, nonerosive arthritis, renal disorder, hematologic disorder, or immunologic disorder and those without. No correlation was revealed between the SLEDAI score and NADH fluorescence.

**Conclusion:**

Changes of NADH fluorescence indicate the reduction in NADH restoration, observed especially during reperfusion, and suggest the occurrence of disorders in the microcirculation of the skin and/or at the mitochondrial level. Such changes of NADH during reperfusion in patients with SLE could be associated with their possible lower sensitivity to hypoxia and possibly with endothelial dysfunction.

## 1. Introduction

Skin manifestations are found in the majority (73–85%) of patients with systemic lupus erythematosus (SLE) [1]. Beside the skin, SLE affects various organs and manifests with different clinical presentations [2]. SLE is a model autoimmune rheumatic disease, and although its pathogenesis is not fully understood, it is thought to arise through an interaction between genetic predisposition and environmental, immunological, and hormonal factors [3]. In SLE, autoimmunity is perpetuated by defective clearance of apoptotic waste and immune complexes, along with disrupted lymphocyte biology and interferon pathways [3]. In addition, atherosclerosis has a great influence on morbidity and mortality in SLE [4]. Risk factors for the development of cardiovascular (CV) disease include both traditional risk factors, such as hyperlipidaemia, hypertension, diabetes, obesity, and smoking, together with SLE-specific factors, such as antiphospholipid antibodies and glucocorticoid therapy [4]. Endothelial function is also impaired: a recent study found that brachial artery endothelium-dependent flow-mediated dilation (baED-FMD), an example of a biophysical marker of endothelial function, was decreased in SLE patients without obvious cardiovascular disease [5]. Another recent study using nailfold videocapillaroscopy (NVC) on nineteen consecutive SLE patients without cardiovascular disease or CV risk factors revealed abnormalities in function and revealed a reduced rate of total CD3+ cells, as well as a higher rate and absolute number of CD3+CD31+CXCR4+ cells [6]. Endothelial dysfunction comprises a systemic disease process involving attenuated endothelium-dependent vasodilation, augmented vasoconstriction, and microvessel structural remodeling throughout the body [7]. Several invasive and noninvasive methods are used in the assessment of endothelial function, such as coronary epicardial vasoreactivity (invasive), coronary microvascular function-Doppler wires (invasive), venous occlusion plethysmography (invasive), flow-mediated vasodilation of brachial artery (noninvasive), finger plethysmography (noninvasive), laser Doppler flowmetry/laser Doppler perfusion monitoring, and laser Doppler imaging (noninvasive); however, the different methods of vascular function assessment are not interchangeable [8, 9].

In recent years, the skin circulation has gained prominence as an accessible and potentially representative vascular bed for examining the mechanisms of microcirculatory function and dysfunction [7]. The very small arterioles and the capillary bed are sites where substrates, such as glucose and oxygen, are supplied by the blood to the tissues and cells [10]. After entering the cells, glucose is degraded in glycolysis and enters the Krebs cycle inside the mitochondria; as a result, the reduced form of nicotinamide adenine dinucleotide (NADH) enters the respiratory chain in the mitochondria, and ATP is synthesized [10]. The final parameter monitored at the microcirculation level is the level of systemic hemoglobin saturation [11]. Unfortunately, monitoring of metabolism at the tissue and cell level is not used in daily clinical practice [11].

The measurement of tissue NADH levels provides the most important information on the metabolic state of the mitochondria in terms of energy production and intracellular oxygen levels [11]. One technique, named flow-mediated skin fluorescence (FMSF), is based on noninvasive, real-time, *in vivo* measurement of NADH fluorescence, emitted from the skin cells of a forearm in response to the blockage and release of blood flow [12]. A pilot study conducted in patients with coronary artery disease (CAD) demonstrated that FMSF offered feasible and reproducible measurement of NADH which was sensitive to acute changes in the skin blood flow and yielded results associated with established plasma endothelial markers [13].

The aim of the study was to examine changes of NADH fluorescence from the epidermis of a forearm measured with the FMSF technique in patients with SLE and to investigate whether they are associated with clinical manifestation of the disease.

## 2. Materials and Methods

The study included a limited number of 36 patients with systemic lupus erythematosus (SLE) (30 women and 6 men) and 34 healthy individuals (25 women and 9 men). The diagnosis of SLE was based on the classification criteria for SLE updated in 1997 by the American College of Rheumatology [14]. The age of the SLE group ranged from 28 to 80 years, with a mean age of 48.8 ± 14.65 years. The mean SLE activity was 4.27 ± 4.34 according to the Systemic Lupus Erythematosus Disease Activity Index (SLEDAI) [15]. The age of the control group ranged from 24 to 69 years, with a mean age of 43.32 ± 15.41 years. There was no statistically significant difference between the mean ages of the SLE patients and the control group. The study was approved by the local Ethics Committee (RNN/71/17/KE), and informed consent was obtained from all participants prior to the study.

**Figure 1:**
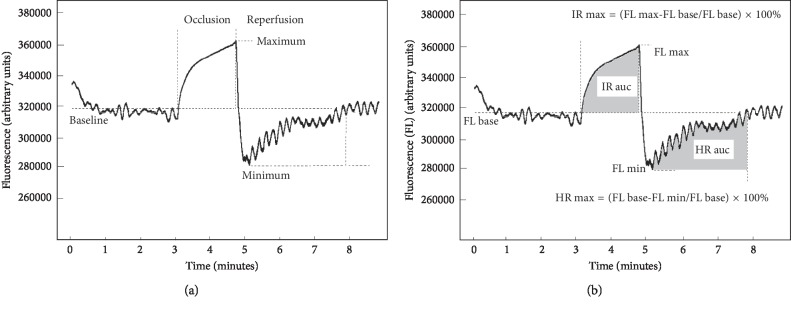
Measurement (a) and definitions (b) of NADH fluorescence emitted from the epidermis of a forearm in a healthy individual in response to the blockage (occlusion) and release of blood (reperfusion) using the flow mediated skin fluorescence (FMSF) technique.

Measurements of NADH were performed with AngioExpert (Angionica, Poland). AngioExpert measures the fluorescence of NADH molecules excited by ultraviolet (UV) radiation of 340 nm (UVB). Since maximum light penetration of 340 nm UVB in the skin is about 0.3 to 0.5 mm, results are determined by fluorescence of NADH within the epidermis [16]. The wavelength of emitted NADH fluorescence (FL) is 460 nm (blue light) [13]. The measurement starts at the rest stage, lasting 1.5 minutes, with the patient in a comfortable, sitting position, and results are displayed as a baseline level (FL base). This is followed by an occlusion (ischaemic) stage, lasting 100 seconds, which is caused by the blockage of blood flow in the arm artery with the sphygmomanometer (blood pressure meter). NADH fluorescence is displayed in arbitrary units (a.u.) and reaches maximum (FL max) in response to ischemia and the accumulation of NADH in keratinocytes. Results are displayed as ischemic response maximum (IR max) and ischemic response area under the curve (IR auc) [17]. Finally, a reperfusion (hyperaemic) stage takes place following sphygmomanometer release: NADH fluorescence rapidly decreases and reaches minimum (FL min) within the first few seconds of reperfusion and then recovers, less steeply, to the baseline level within 4.5 minutes. Results are displayed as hyperemic response max (HR max) and hyperemic response area under the curve (HR auc) [17]. To illustrate, typical results and definitions of measured parameters, obtained in one individual of the healthy control group, are presented in Figure 1.

Statistical analysis was performed with Statistica, version 13 (Statsoft, Poland). The Shapiro–Wilk test did not find the measured parameters to have a normal distribution. Therefore, the nonparametric Mann–Whitney *U* test was used to compare results between two groups and Spearman's rank correlation to measure statistical dependence between two parameters. Results are represented as median with lower (25th) and upper (75th) quartile (25th–75th centile). Age is displayed as mean ± standard deviation of the mean. In all calculations, a *p* value less than 0.05 was regarded as statistically significant.

## 3. Results

The patients' clinical characteristics, according to the classification criteria for SLE updated in 1997 by the American College of Rheumatology, are presented in Table 1.

Changes of NADH fluorescence displayed by ishaemic response maximum (IR max), ischaemic response area under the curve (IR auc), hyperaemic response maximum (HR max), and hyperaemic response area under the curve (HR auc) in patients with systemic lupus erythematosus (SLE) and in the healthy control group are shown in Table 2.

**Figure 2:**
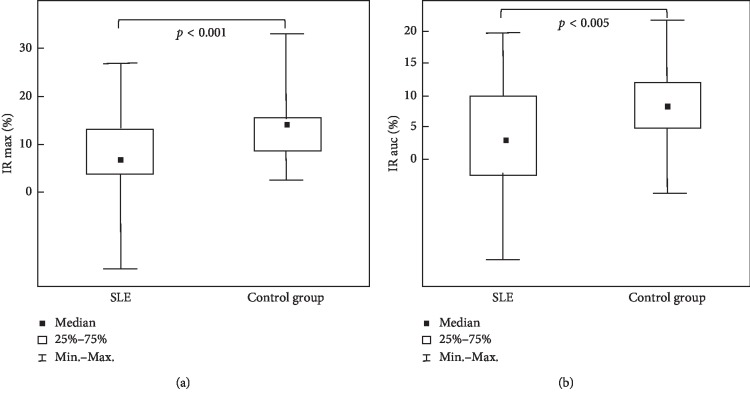
Decreased changes of NADH fluorescence during ischaemic response, represented as IR max (a) and IR auc (b) in patients with SLE compared with the control group.

Changes of NADH fluorescence during ischaemic response, represented as IR max and IR auc, were lower in patients with SLE than those in the control group (*p* < 0.001 and *p* < 0.005, respectively) (Figure 2).

**Figure 3:**
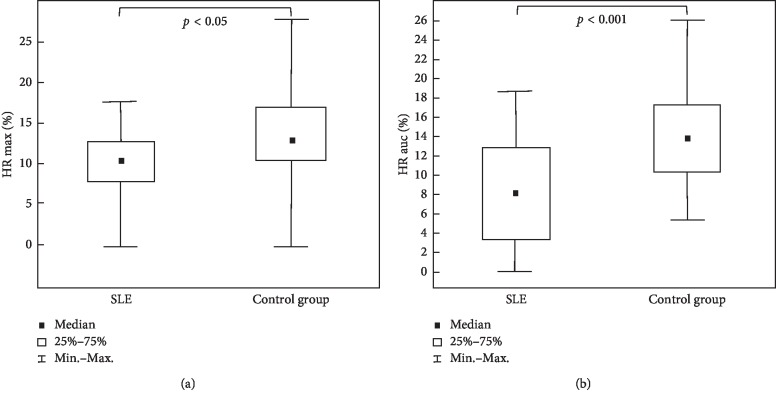
Decreased changes of NADH fluorescence during hyperaemic response, represented as HR max (a) and HR auc (b) in patients with SLE compared with the control group.

Changes of NADH fluorescence during hyperaemic response, represented as HR max and HR auc, were lower in patients with SLE than those in the control group (*p* < 0.05 and *p* < 0.001, respectively) (Figure 3).

**Figure 4:**
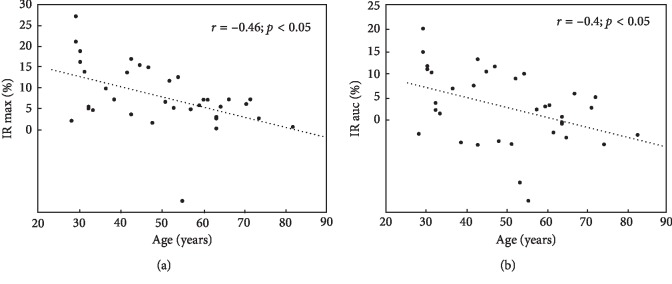
Negative correlation between changes of NADH fluorescence during ischaemic response represented as IR max and IR auc and the age of the patients with SLE.

**Figure 5:**
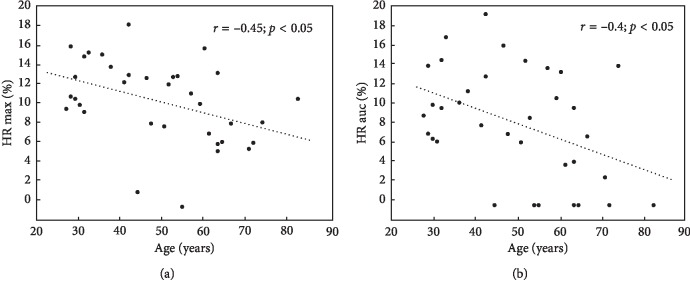
Negative correlation between changes of NADH fluorescence during hyperaemic response represented as HR max and HR auc and the age of the patients with SLE.

All four parameters representing changes of NADH fluorescence during both ischaemic and hyperaemic responses were negatively correlated (*p* < 0.05) with the age of the patients with SLE (Figures 4 and 5).

No difference in changes of NADH fluorescence was found between SLE patients with malar rash, discoid rash, photosensitivity, oral ulcers, nonerosive arthritis, renal disorder, hematologic disorder, and immunologic disorder and those without, with regard to the clinical manifestation of the disease and analysis of each organ involvement separately. No correlation was revealed between the SLEDAI score and NADH fluorescence.

## 4. Discussion

This is the first study to use the noninvasive FMSF technique to evaluate NADH fluorescence in patients with systemic lupus erythematosus (SLE). The key finding of our pilot study is that the changes of NADH were seen to decrease in both ischemic and reperfusion response in SLE patients in comparison to those of the healthy control individuals. Such decreased fluorescence values observed in the epidermis of patients with SLE during blocking and reperfusion of blood flow indicate a reduction in the amount of NADH. All four parameters representing changes of NADH fluorescence were negatively correlated with the age of the patients with SLE, and it should be emphasized that no significant difference in mean age existed between the patient and the control group; hence, the decrease in NADH changes and the correlation with the age observed in our patient group can be extrapolated to other SLE patients.

The reduction of NADH restoration, especially during reperfusion, suggests the occurrence of disorders in the microcirculation of the skin and/or at the mitochondrial level. Under normal conditions, the decrease in oxygen supply associated with arterial occlusion is accompanied by an increase in NADH concentration and NADH fluorescence intensity and is displayed as values of IR max and IR auc in FMSF. Since NADH is a major mitochondrial component which plays a key role in cellular energy metabolism, the measured ischemic response provides an insight into the mitochondrial function, and its amplitude may reflect tissue sensitivity to hypoxia [16]. A recent FMSF study conducted on patients with coronary artery disease (CAD) found IR max and HR max to be decreased in those patients who suffered from left ventricle dysfunction combined with diabetes mellitus, suggesting that microvascular endothelial function is impaired by diseases known to affect the endothelium [16].

The presence of low ischemic response values (IR max and IR auc) in patients with SLE may be due to the fact that they are probably less sensitive to hypoxia. Such a condition could also occur as a result of endothelial dysfunction with links to autoantibodies against double-stranded DNA (dsDNA) and RNA-protein complexes, activation of the type I interferon system, and platelets in SLE patients [18]. Regarding anti-dsDNA antibodies, whose presence constitutes one of the criteria of the American College of Rheumatology (ACR) and Systemic Lupus International Collaborating Clinics for the diagnosis of SLE, their occurrence was found to precede the diagnosis of SLE with a mean onset of 2.7 years before diagnosis, ranging from nine years to less than one month [14, 19, 20]; however, in the present study, no difference in NADH fluorescence was found between patients with immunologic disorder and those without.

The source of the DNA that triggers an immune response in SLE is probably genomic DNA which is released from apoptotic and necrotic cells [21]. It needs to be emphasized that mitochondrial DNA (mtDNA) induces the production of tumor necrosis factor alpha and has been found to display proinflammatory properties [22]. Although mtDNA is known to be extruded by granulocytes, the exact underlying mechanism remains unknown [23]. In SLE, neutrophils were found to extrude DNA when activated with anti-Sm/RNP antibodies in an FcγR, TLR7, and ROS-dependent manner [24]. Antioxidized mtDNA autoantibodies were detected in a fraction of SLE sera [18]. Caielli et al., found that oxidized mtDNA activated plasmacytoid dendritic cells (pDCs) via the TLR9 pathway [18]. In addition, pDCs have been found to play a critical role in the IFN-*α*/*β*-dependent initiation of autoimmune lupus [25]. Hence, it can be suggested that, in contrast to neutrophils from healthy individuals, neutrophils in SLE patients are characterized by aggregation and retention of oxidized mtDNA and overexpress IFN-inducible transcripts. Furthermore, the presence of oxidized mtDNA could induce an immune response and the production of autoantibodies; however, there are no reports of possible extrusion of mtDNA from cells other than neutrophils in SLE, and if such a phenomenon were present, there would be a need to determine whether such mtDNA were a marker of mitochondrial damage in various cell types, especially in patients with SLE. Our findings, therefore, indicate that the mitochondrial metabolism of keratinocytes was altered at the NADH level in patients with SLE, with a reduction of NADH fluorescence being observed during occlusion, followed by the subsequent restoration of its reserve. Nevertheless, it is important to consider two important points when interpreting these findings. Firstly, NADH is a critical substrate for the respiratory chain, and a decrease in its level may lower the oxygen consumption rate and reduce ATP production in cells. Secondly, the role of NADH in cellular metabolism is not only restricted to the respiratory chain, as NADH was found to influence gene transcription through regulation of transcriptional repressor carboxyl-terminal binding protein (CtBP); an increase in NADH level was found to result in gene repression [26]. NAD was found to be involved in the deacetylation of histones, an indicator of genomic silencing [27, 28]. NAD is also considered to be engaged in DNA repair, gene expression, and stress response [28] and as a substrate in the production of (ADP-ribose) polymers on nuclear proteins, such as poly (ADP-ribose) polymerase (PARP), elicited by DNA breaks [29].

The low level of NADH observed in the keratinocytes of patients with SLE suggests that dysregulation of NAD+ and NADH production may also affect other cell types and influence numerous metabolic pathways. On the one hand, low NADH production could reflect disorders of cellular metabolism and on the other hand, reflect endothelial dysfunction and disorders of blood supply. Indeed, endothelial dysfunction is involved in SLE pathogenesis, and contributes to the increased cardiovascular risk observed in SLE patients [30]. Endothelial dysfunction could be defined as a state of impaired endothelial-dependent vasodilatation that also includes endothelial activation: a proinflammatory state with decreased endothelial anticoagulant ability [31]. However, there is a lack of reports focused on the link between endothelial dysfunction and NADH level, and further studies are needed in this regard.

The present study does have some limitations. So far, it is challenging to define whether the measured NADH fluorescence reflects the free-form or protein-bound form of NADH or both. In addition, *in vivo* studies do not distinguish the fraction of NADH that is being examined, i.e., whether it is cytosolic, nuclear, or mitochondrial, and this is also one of the limitations of our study. Research on NAD within the mitochondrial matrix revealed that NAD concentrations were decreased in comparison to the corresponding measurements performed on whole HeLa cells [32]. Nevertheless, our findings support the development of therapeutic efforts aimed at improvement of cellular metabolism in patients with SLE. In a mouse model, supplementation with nicotinamide mononucleotide (NMN) was found to reverse vascular dysfunction [33].

Another limitation is the fact that it enrolled relatively small number of patients with SLE; undoubtedly, further studies conducted on larger populations of patients are required.

Thirdly, more comprehensive data could be provided through a comparison of NADH fluorescence and the amounts of oxidized mtDNA and antioxidized mtDNA antibodies and serum concentrations of proinflammatory cytokines and markers of endothelial dysfunction. However, this will have to await future studies.

## Figures and Tables

**Table 1:** Clinical characteristics of patients with systemic lupus erythematosus (SLE).

Characteristic	Number of patients (%)
Malar rash	27 (75)
Discoid rash	9 (25)
Photosensitivity	29 (80.5)
Oral ulcers	9 (25)
Nonerosive arthritis	31 (86.1)
Pleuritis or pericarditis	0 (0)
Renal disorder	4 (11.1)
Neurologic disorder	0 (0)
Hematologic disorder	28 (77.7)
Immunologic disorder (anti-dsDNA or anti-Sm or antiphospholipid antibodies)	13 (36.1)
Antinuclear antibodies	36 (100)

**Table 2:** Changes of NADH fluorescence in patients with systemic lupus erythematosus (SLE) and in the healthy control group.

Change of NADH fluorescence (%)	SLE		Control group	
	Median	Lower quartile-upper quartile	Median	Lower quartile-upper quartile
IR max	6.75	3.80–13.30	14.25	8.80–15.52
IR auc	3.17	–2.34–9.89	8.48	4.75–12.05
HR max	10.50	7.95–12.75	12.90	10.50–17.00
HR auc	8.17	3.33–12.82	13.91	10.23–17.18

## Data Availability

The digital data used to support the findings of this study are available from the corresponding author upon request.
